# Patient-Reported Vision-Related Quality-of-Life Differences between Primary Angle-Closure Glaucoma and Primary Open-Angle Glaucoma

**DOI:** 10.1371/journal.pone.0163123

**Published:** 2016-09-30

**Authors:** Hui-Chen Cheng, Chao-Yu Guo, Yu-Jing Chen, Mei-Ju Chen, Yu-Chieh Ko, Nicole Huang, Catherine Jui-ling Liu

**Affiliations:** 1 Department of Ophthalmology, Taipei Veterans General Hospital, Taipei, Taiwan; 2 Department of Ophthalmology, School of Medicine, National Yang-Ming University, Taipei, Taiwan; 3 Institute of Public Health, Department of Public Health, National Yang-Ming University, Taipei, Taiwan; 4 Division of Biostatistics, Institute of Public Health, National Yang Ming University, Taipei, Taiwan; 5 Institute of Hospital and Health Care Administration, National Yang-Ming University, Taipei, Taiwan; Bascom Palmer Eye Institute, UNITED STATES

## Abstract

**Purpose:**

To investigate the different impacts on patient-reported vision-related quality of life (pVRQOL) outcomes in patients with primary angle-closure glaucoma(PACG) and primary open-angle glaucoma(POAG).

**Methods:**

Prospective cross-sectional study. PACG and POAG patients who had a best-corrected visual acuity(BCVA) in the better eye equal to or better than 20/60, intraocular pressure controlled at or below 25 mmHg and reliable visual field test were invited to participate. The control group included patients with BCVA in the better eye equal to or better than 20/60 and who did not have major eye disease. A validated Taiwanese version of the 25-item National Eye Institute Visual Function Questionnaire (NEI VFQ-25(T)) was performed to assess pVRQOL. The association between each domain of NEI VFQ-25(T) among 3 groups was determined using multivariable linear regression analysis.

**Results:**

A total of 106 PACG, 186 POAG, and 95 controls were enrolled. In multivariable regression analysis of all three groups(PACG/POAG/controls), compared to POAG, PACG showed a weakly positive association with social functioning (R^2^ = 0.13, β = 0.22, *P* = 0.04). PACG showed no significantly negative impact on pVRQOL compared to controls. Taking only glaucoma patients into consideration, PACG patients had a higher score on social functioning compared to POAG (R^2^ = 0.16, β = 0.27, *P* = 0.01). The results of other domains of NEI VFQ-25(T) between the two groups did not differ significantly(p>0.05).

**Conclusions:**

In patients with controlled disease, the impact of PACG and POAG on most domains of NEI VFQ-25(T) were similar, except for better social functioning in PACG compared to POAG.

## Introduction

Primary angle-closure glaucoma (PACG) is one of the major types of glaucoma worldwide, with the highest prevalence in Asia,[1, 2] and it is reported that over 80% of PACG patients are of Asian ethnicity.[3] These patients have a crowded anterior segment and live with the risk of an acute attack of angle closure, which is different from primary open-angle glaucoma (POAG). [4–7]

Several population studies have shown that glaucoma patients might have difficulties in daily-life activities and patient-reported vision-related quality of life (pVRQOL).[8–21] However, most of these studies focused on POAG patients, and only rare reports have investigated pVRQOL in PACG patients. Wang et al. evaluated 108 POAG and 84 PACG patients and found no significant difference in glaucoma functioning score as assessed by the Glaucoma Quality of Life-15 questionnaire using univariate analysis.[22] However, different conclusions are possible when a study does not adjust for several relevant non-ocular factors for pVRQOL, such as cognitive ability, comorbid illness, education, and race.[8, 23] Considering that POAG and PACG have different mechanisms and clinical presentations, we hypothesized that except for the well-recognized visual acuity (VA) and visual field (VF) loss, PACG and POAG per se may have different influences on pVRQOL.

In this prospective study, we aimed to investigate the different impact of PACG and POAG on pVRQOL outcomes in Chinese patients while taking potential confounding factors into consideration.

## Materials and Methods

This prospective cross-sectional study was conducted from March 2012 to January 2013 in Taipei Veterans General Hospital (VGH). Eligible consecutive glaucoma patients and control subjects were invited to participate. The study protocol adhered to the tenets of the Declaration of Helsinki and was approved by the institutional review board of Taipei Veterans General Hospital before the study began. Written informed consent was obtained from each patient after the aims and methods of the study had been fully explained.

Patients with POAG/PACG had to fulfill the following criteria before enrollment: (1) best-corrected VA (BCVA) in the better eye equal to or better than 20/60 in order to avoid the effect of low vision on pVRQOL[24]; (2) intraocular pressure (IOP) controlled at or below 25 mmHg; and (3) a reliable VF test within 3 months of enrollment, defined as fixation loss ≤30%, false positive ≤15%, and false negative ≤15%. The diagnosis of POAG/PACG was made by glaucoma specialists (CJlL, YCK, or MJC) according to corresponding glaucomatous optic disc changes, reproducible VF defects in a retinal nerve fiber bundle pattern, a normal open angle on gonioscopy examination in POAG, and a closed angle before laser treatment or cataract extraction in PACG. A glaucoma hemifield test result outside normal limits or a pattern standard deviation outside 95% of age-specific normal limits defined a glaucomatous VF defect. The limitation of IOP was applied because we aimed to investigate the impact of different disease nature in PACG/POAG and tried to eliminate the influence of uncontrolled disease on pVRQOL.

The control group included patients with BCVA in the better eye equal to or better than 20/60 and who were undergoing routine physical examinations or follow-up for diseases other than corneal disorder, retinal disease, optic neuropathy, glaucoma, or other pathology known to affect quality of life (QOL) except for cataract in Taipei VGH.

We excluded patients with histories of the following to avoid a confounding effect on pVRQOL, including a history of ocular surgeries within 3 months before recruitment, a history of laser refractive surgery, concurrent ocular disease, neurological disease that could cause a VF defect, or severe comorbidities known to affect QOL, such as dementia, end-stage renal disease, dysfunctional Parkinsonism, or systemic malignancy.[25, 26]

All participants received a review of medical history, assessment of binocular habitual VA, BCVA, slitlamp biomicroscopy, tonometry, and dilated ophthalmoscopy. The glaucoma patients received additional gonioscopy and standard achromatic automated perimetry with the 24–2 Swedish Interactive Threshold Algorithm standard of the Humphrey Field Analyzer 750i (version 4.2, Humphrey Instruments, San Leandro, CA). Measured VA was then converted to the logarithm of the minimum angle of resolution (logMAR) values for further analysis. The results of VF were converted into integrated VF (IVF) using the “best sensitivity” method.[27] From each pair of spatially corresponding points of two eyes, the better original sensitivity (dB) and total deviation (TD) value was selected for indicating the sensitivity and TD of the particular locus in binocular vision. The two most nasal points in each eye were discarded. The mean deviation (MD) of IVF was calculated as the mean of the individual binocular TD values.

### Socioeconomic and clinical characteristics questionnaire

At the time of enrollment, a standardized questionnaire regarding socioeconomic and clinical information was obtained. The history of ocular and medical diseases was checked by self-reported method, including ophthalmic surgery, glaucoma medication class and dosing frequency, asthma/chronic obstructive pulmonary disease, coronary artery disease, diabetes mellitus, hypertension, hyperlipidemia, hypotension, migraine, liver disease, Raynaud’s phenomenon, and renal disease. Psychological disorders, which were diagnosed by doctors with medication prescribed, were also recorded, including anxiety, depression, drug addiction/alcoholism, and insomnia. The socioeconomic status was defined as education level (none or elementary school, junior or senior high school, college or research institute), marriage status (single or married), living status (alone or living with company), career (retired or working), and monthly salary (0, >0–<60,000 or ≥60,000 in new Taiwan dollars).

### 25-item National Eye Institute Visual Function Questionnaire (NEI VFQ-25) in a validated Taiwanese version

Each patient completed a validated Taiwanese version of the NEI VFQ-25 (NEI VFQ-25 (T)) with the help of a trained assistant to evaluate the pVRQOL.[28, 29] The questionnaire contained 25 vision-related questions which were categorized into 11 vision-targeted domains.[28] An additional question regarding the patient’s general health was also recorded.[28] A composite score was obtained by averaging the scores of 11 vision-targeted domains.[28] The score of each domain ranged from 1 to 100, with a higher score representing better pVRQOL.[28]

### Statistical analysis

The continuous variables are presented as means (±standard deviation), and categorical variables are presented as numbers (%). For converting the scores into a normal distribution, the scores of each domain of the NEI VFQ-25(T) were skewed using the SAS procedure PROC RANK, which computes normal scores from the ranks of original values.

In the beginning, univariate analysis was used to evaluate the association between each demographic, clinical, and socioeconomic variable and each normalized score of the NEI VFQ-25(T). The subsequent multivariable linear regression analysis had two models, and the outcomes were defined as each normalized score of the NEI VFQ-25(T). The first one included participants from all three groups (PACG/POAG/control), and the second one focused on patients with PACG and POAG. Variables with a *P* value < 0.1 in the univariate analysis were adjusted in the multivariable linear regression model. The level of significance for the univariate screening regressions was set at p<0.1 because more stringent significance levels may lead to the exclusion of potentially useful predictor variables. In addition, age, gender, habitual VA, and IVF MD (only in the second model of POAG/PACG) were forced to be part of the analysis because previous studies have shown that these factors have significant impact on health-related quality of life.[15, 21, 30–32] The IVF MD was not put into the first model because the control subjects did not receive VF examinations and the data were missing. To avoid type II error, we present the original R^2^, β, and *P* values of each variable in different models, instead of *P* values corrected with Bonferroni’s method.

All statistical analyses were performed with commercially available software (SAS, ver. 9.2; SAS Institute Inc., Cary, NC).

## Results

A total of 394 consecutive patients were eligible for this study, but three patients declined to participate, and four patients were excluded because of two unreliable VF tests after enrollment. Thus, a total of 387 patients were enrolled, including 106 patients with PACG, 186 patients with POAG, and 95 control subjects. The mean age was 64.7 years (range, 19–91 years) among all participants, and 55.6% of them were male. Patient demographic and socioeconomic data are listed in Table 1, and patient ophthalmologic characteristics are presented in Table 2. The patients with PACG were older (*P* < 0.0001) and had worse binocular VA (*P* = 0.0008) and a higher proportion of hypertension (*P* = 0.02), coronary artery disease (*P* = 0.005), and insomnia (*P* = 0.02) compared to other groups. Moreover, PACG patients had a longer disease duration (*P* = 0.007) and a higher proportion receiving laser treatment (*P* < 0.0001) compared to POAG patients. The results of multivariable analysis are shown in S1 and S2 Tables. We found that a higher education level (R^2^ = 0.18, β = 0.35, *P* = 0.03), no coronary artery disease (R^2^ = 0.18, β = -0.39, *P* = 0.05), no insomnia (R^2^ = 0.18, β = -0.59, *P* = 0.002), and better habitual binocular VA (in logMAR, R^2^ = 0.18, β = -2.49, *P* ≤ 0.001) correlated with higher composite score. When the analysis included only PACG and POAG patients, IVF MD was an additional factor that correlated positively with composite score (R^2^ = 0.21, β = 0.04, *P* = 0.002). Our results showed that the composite score increased by 0.04 units with each 1 dB improvement in MD of IVF (β = 0.04). In addition, 21% of the variation of the composite score could be explained by MD for the full-field IVF (R^2^ = 0.21).

**Table 1:** Demographic and socioeconomic data in patients with primary angle-closure glaucoma, primary open-angle glaucoma, and control subjects.

	Controls (n = 95)		POAG (n = 186)		PACG (n = 106)	
Age, mean (SD)[range]^‡^	66.6 (15.5) [22–90]		59.1 (13.1) [19–86]		72.8 (9.0) [51–91]	
**Sex, No. (%)**^‡^						
** Men**	34	(35.8)	131	(70.4)	50	(47.2)
**Education, No. (%)**^‡^						
** None or elementary school**	25	(26.3)	18	(9.8)	30	(28.3)
** Junior or senior high school**	39	(41.1)	69	(37.5)	43	(40.6)
** College or research institute**	31	(32.6)	97	(52.7)	33	(31.1)
**Marriage, No. (%)**						
** Single**	9	(9.5)	17	(9.1)	5	(4.7)
** Married**	86	(90.5)	169	(90.9)	101	(95.3)
**Living status, No. (%)**						
** Alone**	8	(8.4)	13	(7.0)	9	(8.5)
** Living with company**	87	(91.6)	173	(93.0)	97	(91.5)
**Career, No. (%)**^‡^						
** Retired**	71	(74.7)	92	(49.7)	95	(91.3)
** Working (including student)**	24	(25.3)	93	(50.3)	9	(8.7)
**Monthly salary (in NTD), No. (%)**^‡^						
** 0**	70	(73.7)	83	(45.6)	86	(81.9)
** >0–<60,000**	10	(10.5)	42	(23.1)	15	(14.3)
** ≥60,000**	15	(15.8)	57	(31.3)	4	(3.8)
**Medical comorbidities**^§^**, No (%)**						
Hypertension*	37	(38.9)	55	(29.6)	48	(45.3)
** Hyperlipidemia**	11	(11.6)	28	(15.1)	15	(14.2)
** Diabetes mellitus**	13	(13.7)	22	(11.8)	14	(13.2)
** Hypotension**	0	(0.0)	4	(2.2)	0	(0.0)
** Migraine**	0	(0.0)	1	(0.5)	0	(0.0)
** Coronary artery disease**^†^	9	(9.5)	6	(3.2)	14	(13.2)
** Asthma/COPD**	2	(2.1)	4	(2.2)	5	(4.7)
** Liver disease**	1	(1.1)	5	(2.7)	4	(3.8)
** Renal disease**	3	(3.2)	2	(1.1)	2	(1.9)
**Psychological comorbidities**^||^**, No. (%)**						
** Depression**	3	(3.2)	1	(0.5)	2	(1.9)
Insomnia*	7	(7.4)	9	(4.8)	15	(14.2)

Abbreviations: COPD, chronic obstructive pulmonary disease; NTD, new Taiwan dollars; PACG, primary angle-closure glaucoma; POAG, primary open-angle glaucoma; SD, standard deviation

The differences among three groups were tested by analysis of variance (ANOVA) for continuous variables and chi-square test for categorical variables.

* *P* < 0.05;

^†^ *P* < 0.01;

^‡^ *P* < 0.001.

^§^ None of our patients had Raynaud’s phenomenon.

^||^ None of our patients had anxiety disorder or drug/alcohol addiction.

**Table 2:** Ophthalmologic characteristics in patients with primary angle-closure glaucoma, primary open-angle glaucoma, and control subjects.

	Controls (n = 95)		POAG (n = 186)		PACG (n = 106)	
Binocular habitual visual acuity (LogMAR), mean (SD) [range]*	0.04 (0.08)	[-0.3–0.3]	0.04 (0.10)	[-0.18–0.70]	0.08 (0.11)	[-0.18–0.40]
Spectacles dependent, No. (%)*	66	(69.5%)	167	(89.8%)	84	(79.2%)
**Spherical equivalent (diopter), mean (SD) [range]**						
** Right eye**^†^	-1.25 (2.33)	[-10.38–2.75]	-3.60 (3.70)	[-14.38–3.00]	-0.30 (1.83)	[-7.25–3.63]
** Left eye** ^†^	-1.12 (2.27)	[-10.25–2.50]	-3.66 (3.52)	[-13.25–3.38]	-0.15 (1.58)	[-5.13–4.00]
**Central corneal thickness (μm), mean (SD) [range]**			559.2 (37.6)	[468–648]	558.2 (35.4)	[470–659]
**Intraocular pressure (mmHg), mean (SD) [range]**						
** Right eye**	14.2 (2.8)	[8–24]	14.84 (2.87)	[8–23]	14.59 (2.74)	[8–21]
** Left eye**	14.7 (2.8)	[7–22]	14.91 (2.83)	[7–24]	14.68 (2.86)	[9–21]
**Lens status, No. (%)**^†^^,^^‡^						
** Clear lens OU**	10	(10.5%)	39	(21.0%)	0	(0.0%)
** Cataract**	15	(15.8%)	108	(58.1%)	51	(48.1%)
** Pseudophakia**	70	(73.7%)	39	(21.0%)	55	(51.9%)
**History of ophthalmic surgery, No. (%)**^†^	72	(75.8%)	75	(40.3%)	62	(58.5%)
**Disease status of glaucoma**						
Disease duration (months), mean (SD) [range]*			81.8 (69.1)	[1–404]	106.4 (83.9)	[1–405]
** No. of current glaucoma drug, mean (SD) [range]**			1.8 (0.9)	[0–4]	1.75 (1.0)	[0–4]
** Dosing frequency of glaucoma medication (times/day), mean (SD) [range]**			2.0 (1.1)	[0–5]	2.0 (1.1)	[0–5]
** Trabeculoplasty/LPI, No. (%)**^†^			30	(16.1%)	93	(87.7%)
** Trabeculectomy, No. (%)**			47	(25.3%)	35	(33.0%)
** Needling or revision surgery, No. (%)**			26	(14.0%)	10	(9.4%)
**MD of the whole IVF (dB), mean (SD) [range]**			-4.84 (4.76)	[-27.56–2.17]	-5.38 (4.83)	[-24.12–1.62]

Abbreviations: dB, decibel; IVF, integrated visual field; LogMAR, logarithm of the minimum angle of resolution; LPI, laser peripheral iridotomy; MD, mean deviation; OU, bilateral eyes; PACG, primary angle-closure glaucoma; POAG, primary open-angle glaucoma; SD, standard deviation

The differences among three groups were tested by analysis of variance for continuous variables and chi-square test for categorical variables.

* *P* < 0.01;

^†^ *P* < 0.001.

^‡^ Cataract means the patient had either binocular cataract or monocular cataract and clear lens in the other eye. Pseudophakia means the patient had at least one eye in the pseudophakic status.

In the first model, all three groups (POAG/PACG/control) were analyzed. The multivariable linear regression analysis showed that PACG patients had better social functioning score compared to POAG (R^2^ = 0.13, β = 0.22, *P* = 0.04). In addition, PACG patients had a higher score for distant activity as compared to control subjects (R^2^ = 0.18, β = 0.43, *P* = 0.02) (Table 3).

**Table 3:** Results of the multivariable linear regression model for selected scores on the NEI-VFQ 25 in patients with primary angle-closure glaucoma, primary open-angle glaucoma, and control subjects.

	General vision		Near activities		Distant activities		Social functioning		Dependency		Driving		Peripheral vision		Composite scores	
	β	p	β	p	β	p	β	p	β	p	β	p	β	p	β	p
**PACG/controls**	-0.24	0.10	0.26	0.16	0.43	0.02	0.17	0.26	0.21	0.20	-0.14	0.51	-0.12	0.35	0.22	0.26
**PACG/POAG**	0.10	0.42	0.23	0.10	0.06	0.63	0.22	0.04	0.05	0.66	0.24	0.17	0.09	0.44	0.20	0.14
**Age (years)**	0.01	0.04	0.01	0.04	-0.0001	0.98	0.005	0.11	0.01	0.30	-0.01	0.13	0.005	0.17	0.008	0.07
**Gender (M/W)**	-0.13	0.23	-0.11	0.36	0.20	0.06	0.05	0.55	0.07	0.47	0.40	0.01	0.18	0.04	0.15	0.19
**Education**																
Junior or senior high school/none or elementary school	0.03	0.81	0.13	0.39					0.10	0.43					0.18	0.21
Above college/none or elementary school	0.19	0.19	0.27	0.11					0.18	0.17					0.35	0.03
**Coronary artery disease (yes/no)**							-0.25	0.11	-0.25	0.13	-0.29	0.37			-0.39	0.05
**Asthma/COPD (yes/no)**					-1.49	0.01										
**Insomnia (yes/no)**	-0.46	0.009	-0.33	0.07	-0.63	<0.001	-0.21	0.15	-0.42	0.009			-0.36	0.02	-0.59	0.002
**Dosing frequency of glaucoma medication (times/day)**			0.01	0.90	-0.14	0.16	-0.07	0.09	-0.10	0.03					-0.10	0.36
**Binocular habitual visual acuity (LogMAR)**	-2.41	<0.001	-1.51	0.008	-0.99	0.05	-2.19	<0.001	-2.44	<0.001	-2.01	0.01	-1.36	0.003	-2.49	<0.001
**R-squared**	0.15		0.15		0.18		0.13		0.17		0.20		0.12		0.18	

Abbreviations: COPD, chronic obstructive pulmonary disease; LogMAR, logarithm of the minimum angle of resolution; M, men; NEI VFQ-25, National Eye Institute Visual Function Questionnaire; PACG, primary angle-closure glaucoma; POAG, primary open-angle glaucoma; W, women

This model was adjusted for age, sex, binocular visual acuity, and other factors regarding socioeconomic data and ophthalmologic characteristics that attained statistical significance during univariate analysis. The factors that did not have statistical significance in the univariate analysis were not analyzed in the regression model, and the columns were empty.

In the second model, considering patients with PACG and POAG, only a significantly positive association for social functioning was noted in the PACG group compared to POAG patients (R^2^ = 0.16, β = 0.27, *P* = 0.01) (Table 4).

**Table 4:** Results of the multivariable linear regression model for selected scores on the NEI-VFQ 25 in patients with primary angle-closure glaucoma and primary open-angle glaucoma.

	General vision		Near activities		Distant activities		Social functioning		Dependency		Driving		Peripheral vision		Composite scores	
	β	p	β	p	β	p	β	p	β	p	β	p	β	p	β	p
**PACG/POAG**	0.04	0.74	0.19	0.17	0.02	0.86	0.27	0.01	0.03	0.77	0.12	0.50	0.07	0.53	0.14	0.32
**Age (years)**	0.02	0.003	0.01	0.15	0.001	0.85	0.002	0.54	0.005	0.36	-0.02	0.10	0.005	0.27	0.01	0.03
**Gender (M/W)**	-0.10	0.38	-0.13	0.32	0.17	0.15	0.08	0.44	0.10	0.35	0.38	0.04	0.27	0.008	0.14	0.24
**Education**																
Junior or senior high school/none or elementary school	0.02	0.90	-0.10	0.58					0.05	0.69					0.18	0.27
Above college/none or elementary school	0.20	0.23	0.07	0.71					0.10	0.50					0.34	0.05
**Coronary artery disease (yes/no)**							-0.39	0.04	-0.21	0.25	-0.39	0.28			-0.37	0.09
**Asthma/COPD (yes/no)**					-1.57	0.01										
**Insomnia (yes/no)**	-0.41	0.04	-0.37	0.07	-0.53	0.006	-0.20	0.25	0.35	0.04			-0.14	0.43	-0.47	0.02
**Dosing frequency of glaucoma medication (times/day)**			0.02	0.87	-0.15	0.14	-0.06	0.13	-0.07	0.11					-0.12	0.23
**Binocular habitual visual acuity (LogMAR)**	-2.33	<.0001	-1.11	0.06	-0.85	0.13	-2.24	<.0001	-2.34	<.0001	-1.27	0.13	-1.10	0.02	-2.21	0.0002
**MD of the whole IVF (dB)**	0.02	0.06	0.04	0.005	0.02	0.11	0.01	0.35	0.04	<.0001	0.05	0.0002	0.04	<.0001	0.04	0.002
**R-squared**	0.14		0.21		0.18		0.16		0.24		0.24		0.17		0.21	

Abbreviations: COPD, chronic obstructive pulmonary disease; dB, decibel; IVF, integrated visual field; LogMAR, logarithm of the minimum angle of resolution; M, men; MD, mean deviation; NEI VFQ-25, National Eye Institute Visual Function Questionnaire; PACG, primary angle-closure glaucoma; POAG, primary open-angle glaucoma; W, women

This model was adjusted for age, sex, binocular visual acuity, MD of the whole IVF, and other factors regarding socioeconomic data and ophthalmologic characteristics that attained statistical significance during univariate analysis. The factors that did not have statistical significance in the univariate analysis were not analyzed in the regression model, and the columns were empty.

## Discussion

In this prospective cross-sectional study of 106 Chinese patients with PACG, 186 patients with POAG, and 95 control subjects, we found that PACG was positively associated with the domain of social functioning compared to POAG. Additionally, PACG had a positive correlation with distant activities compared to the controls. The other domains of NEI VFQ-25(T) among 3 groups were similar. When only PACG and POAG patients were included in the regression model, PACG showed a positive impact on social functioning compared to POAG.

PACG comprises a major proportion of glaucoma in Asia, but few investigations of pVRQOL have focused on these patients.[22] Wang et al. found no significant difference for glaucoma functioning score, as assessed by the Glaucoma Quality of Life-15 questionnaire, on univariate analysis (*P* = 0.61) of data for 108 POAG patients and 84 PACG patients.[22] Kong et al. investigated 50 PACG patients, 50 POAG patients, and 50 normal controls and reported that the level of anxiety and depression was higher in PACG than in POAG and controls, using analysis of variance.[33] Their findings were the result of univariate analysis and not adjusted for other factors. As we know, several relevant factors, such as age, VA, VF defect, cognitive ability, comorbid illness, education, and race, may impact pVRQOL significantly,[8, 21, 23, 30] so a cautious interpretation of these conclusions is required in the absence of multivariable adjustment.

In our first model, the impact of PACG, POAG and control group on pVRQOL was generally similar except for a positively correlation with distant activities in PACG compared to the controls (Table 3 and S1 Table). As we had mentioned before, we set limitation on IOP to avoid the influence of uncontrolled disease. Moreover, 87 percent of our PACG patients received laser peripheral iridotomy, and all others except one patient received cataract surgery, which helped to optimize IOP control.[4–7] These patients were properly treated and had an understanding of their disease condition, which may have improved their satisfaction on pVRQOL. Our results suggested that glaucoma patients with controlled disease might have comparable QOL to control subjects.

Earlier studies have found that physical activity did not differ significantly between individuals with and without glaucoma but identified a dose response relating VF defect to decreased activity.[34, 35] In our analysis, we could not delineate the impact of VF in the first model because of the lack of VF data in control subjects. We speculated that PACG patients might still have good distant activity under stable disease status; however, safety remains a major concern because these patients may have a higher risk of falls and bumping into objects.[10]

In our study, PACG patients had better social functioning than POAG patients on multivariable linear regression analysis, and this result was consistent in both of the regression models after adjusting several relevant factors including VA and VF. The two diseases are different in nature: POAG patients commonly have no symptoms until an advanced stage whereas PACG patients usually suffer from eye pain, suddenly blurred vision, and even acute attacks. However, timely and proper treatment may relieve these symptoms.[4–7] The questions regarding social functioning in NEI VFQ-25 were” Because of your eyesight, how much difficulty do you have seeing how people react to things you say?” and “Because of your eyesight, how much difficulty do you have visiting with people in their homes, at parties, or in restaurants?”.[28] Several studies have shown that glaucoma patients may experience more anxiety and depression and be more withdrawn and introverted.[33, 36–38] In our study, most of the POAG patients were of working age (mean age, 59.1 years in POAG vs. 72.8 years in PACG), and the psychological burden may be more prominent in this population compared to PACG. In addition, POAG patients are usually asymptomatic before they are diagnosed and treated, and the subsequent treatment causes inconvenience and ocular discomfort, in contrast to PACG patients, who may experience some improvement after laser treatment or lens extraction.[4–7] In patients with controlled disease, the psychological burden and relative disappointment may contribute to the unfavorable response to the questions regarding social functioning in POAG patients. Proper communication, psychological support, and understanding of the disease course may help to improve these patients’ QOL.

In our study, patients with PACG had lower education level and income than POAG patients, which was consistent with other study.[39] Lower education level with less myopia and lower income with undernutrition may contribute to the susceptibility to PACG.[39–41] Conversely, higher socioeconomic status with increased prevalence of myopia and eye care visits may increase patients’ susceptibility to POAG.[39, 40, 42]

This study has several strengths. The glaucoma was diagnosed by glaucoma specialists with a regular follow-up, which reflects the real-world situations of glaucoma patients. We tried to clarify the impact of and differences in PACG and POAG on pVRQOL and provide meaningful advice to patients and their caregivers. In addition, we took into consideration many known and relevant factors reported to influence pVRQOL to avoid confounding effects and to delineate the real association.

Nevertheless, our study findings should be interpreted in the context of the following limitations. The sample size was relatively small and imbalanced among groups, reflecting the difficulties of a hospital-based clinical study. The diagnosis of POAG/PACG was made by 3 different glaucoma specialists (CJlL, YCK, or MJC), which may possess the possibility of observer’s bias. However, we had standardized the glaucoma diagnostic algorithm among the glaucoma specialists to minimize the likelihood of observer’s bias. The history of ocular and medical diseases was self-reported though the questionnaire, which may have the possibility of recall bias. Conform to our daily practice, patients without glaucoma will not receive the examination of central corneal thickness (CCT). Therefore, our control subjects did not have the data of CCT and CCT was not put into our first model regarding control/POAG/PACG patients. We used IVF to simulate binocular VF. Although IVF may not be fully representative of true VF with both eyes open, it correlates well with other binocular tests and assessment of vision.[27, 43, 44] Finally, the control subjects were invited from among hospital-based patients, who differ from healthy controls; however, we performed comprehensive ophthalmological examinations and surveys of medical history to avoid including control subjects with QOL confounders.

In conclusion, PACG and POAG in controlled status may have similar impacts on most domains of NEI VFQ-25(T), except for better social functioning in PACG patients compared to POAG. With proper management and good patient education, patients with PACG and POAG could still have a pVRQOL that is comparable to people without glaucoma.

## Supporting Information

S1 Table Results of the multivariable linear regression model for each score on the NEI-VFQ 25 in patients with primary angle-closure glaucoma, primary open-angle glaucoma, and control subjects. (DOC)

S2 Table Results of the multivariable linear regression model for each score on the NEI-VFQ 25 in patients with primary-angle closure glaucoma and primary open-angle glaucoma. (DOC)
