# Supplementary material for: Patient-Reported Vision-Related Quality-of-Life Differences between Primary Angle-Closure Glaucoma and Primary Open-Angle Glaucoma
Source: PLoS One. 2016 Sep 30;11(9):e0163123. doi: 10.1371/journal.pone.0163123 (PMC5045164; doi:10.1371/journal.pone.0163123)
Supplement: S1 Table — (DOC) [file pone.0163123.s001.doc]

**S1 Table. Results of the multivariable linear regression model for each score on the NEI-VFQ 25 in patients with primary angle-closure glaucoma, primary open-angle glaucoma, and control subjects**

|  | **General health** | | **General vision** | | **Ocular pain** | | **Color vision** | | **Near activities** | | **Distant activities** | | **Social functioning** | | **Role difficulties** | | **Mental health** | | **Dependency** | | **Driving** | | **Peripheral vision** | | **Composite scores** | |
| --- | --- | --- | --- | --- | --- | --- | --- | --- | --- | --- | --- | --- | --- | --- | --- | --- | --- | --- | --- | --- | --- | --- | --- | --- | --- | --- |
|  | β | p | β | p | β | p | β | p | β | p | β | p | β | p | β | p | β | p | β | p | β | p | β | p | β | p |
| **PACG/controls** | -0.17 | 0.23 | -0.24 | 0.10 | 0.34 | 0.06 | -0.001 | 0.99 | 0.26 | 0.16 | 0.43 | 0.02 | 0.17 | 0.26 | -0.04 | 0.81 | -0.14 | 0.47 | 0.21 | 0.20 | -0.14 | 0.51 | -0.12 | 0.35 | 0.22 | 0.26 |
| **PACG/POAG** | 0.15 | 0.26 | 0.10 | 0.42 | 0.10 | 0.47 | 0.03 | 0.67 | 0.23 | 0.10 | 0.06 | 0.63 | 0.22 | 0.04 | 0.15 | 0.23 | -0.01 | 0.93 | 0.05 | 0.66 | 0.24 | 0.17 | 0.09 | 0.44 | 0.20 | 0.14 |
| **Age (years)** | 0.005 | 0.46 | 0.01 | 0.04 | 0.007 | 0.25 | -0.001 | 0.55 | 0.01 | 0.04 | -0.0001 | 0.98 | 0.005 | 0.11 | 0.006 | 0.13 | 0.01 | 0.01 | 0.01 | 0.30 | -0.01 | 0.13 | 0.005 | 0.17 | 0.008 | 0.07 |
| **Gender (men/women)** | 0.04 | 0.70 | -0.13 | 0.23 | 0.35 | 0.002 | 0.02 | 0.72 | -0.11 | 0.36 | 0.20 | 0.06 | 0.05 | 0.55 | 0.20 | 0.06 | 0.19 | 0.09 | 0.07 | 0.47 | 0.40 | 0.01 | 0.18 | 0.04 | 0.15 | 0.19 |
| **Socioeconomic data** | | | | | | | | | | | | | | | | | | | | | | | | | | |
| **Education** |  |  |  |  |  |  |  |  |  |  |  |  |  |  |  |  |  |  |  |  |  |  |  |  |  |  |
| Junior or senior high school/none or elementary school | 0.58 | <.0001 | 0.03 | 0.81 |  |  |  |  | 0.13 | 0.39 |  |  |  |  |  |  | 0.26 | 0.07 | 0.10 | 0.43 |  |  |  |  | 0.18 | 0.21 |
| Above college/none or elementary school | 0.71 | <.0001 | 0.19 | 0.19 |  |  |  |  | 0.27 | 0.11 |  |  |  |  |  |  | 0.27 | 0.09 | 0.18 | 0.17 |  |  |  |  | 0.35 | 0.03 |
| **Marriage (married/single)** | -0.18 | 0.39 |  |  |  |  |  |  | -0.19 | 0.40 |  |  |  |  |  |  |  |  | -0.31 | 0.09 | -0.10 | 0.69 |  |  |  |  |
| **Career (working/none)** | -0.28 | 0.18 |  |  | -0.12 | 0.59 |  |  |  |  |  |  |  |  |  |  |  |  |  |  | -0.11 | 0.66 |  |  |  |  |
| **Monthly salary (in new Taiwan dollars)** |  |  |  |  |  |  |  |  |  |  |  |  |  |  |  |  |  |  |  |  |  |  |  |  |  |  |
| <60,000/0 | 0.49 | 0.01 |  |  | 0.03 | 0.88 |  |  |  |  | -0.29 | 0.04 |  |  |  |  |  |  |  |  | -0.13 | 0.59 |  |  |  |  |
| ≥60,000/0 | 0.71 | 0.001 |  |  | -0.07 | 0.75 |  |  |  |  | -0.41 | 0.01 |  |  |  |  |  |  |  |  | -0.02 | 0.93 |  |  |  |  |
| **Medical comorbidities** | | | | | | | | | | | | | | | | | | | | | | | | | | |
| **Hypertension** | -0.07 | 0.51 |  |  |  |  |  |  | -0.06 | 0.60 |  |  |  |  |  |  |  |  | -0.03 | 0.75 |  |  |  |  | -0.04 | 0.70 |
| **Coronary artery disease** | -0.47 | 0.01 |  |  |  |  |  |  |  |  |  |  | -0.25 | 0.11 |  |  |  |  | -0.25 | 0.13 | -0.29 | 0.37 |  |  | -0.39 | 0.05 |
| **Migraine** | -2.20 | 0.01 |  |  |  |  |  |  |  |  |  |  |  |  |  |  |  |  |  |  |  |  |  |  |  |  |
| **Asthma/COPD** | -1.09 | 0.08 |  |  |  |  |  |  |  |  | -1.49 | 0.01 |  |  |  |  |  |  |  |  |  |  |  |  |  |  |

**S1 Table. Results of the multivariable linear regression model for each score on the NEI-VFQ 25 in patients with primary angle-closure glaucoma, primary open-angle glaucoma, and control subjects (continued)**

|  | **General health** | | **General vision** | | **Ocular pain** | | | **Color vision** | | **Near activities** | | **Distant activities** | | | **Social functioning** | | **Role difficulties** | | **Mental health** | | **Dependency** | | **Driving** | | **Peripheral vision** | | **Composite scores** | | |
| --- | --- | --- | --- | --- | --- | --- | --- | --- | --- | --- | --- | --- | --- | --- | --- | --- | --- | --- | --- | --- | --- | --- | --- | --- | --- | --- | --- | --- | --- |
|  | β | p | β | p | β | | p | β | p | β | p | β | | p | β | p | β | p | β | p | β | p | β | p | β | p | β | p | |
| **Psychological comorbidities** | | | | | | | | | | | | | | | | | | | | | | | | | | | | | |
| **Insomnia** | -0.14 | 0.44 | -0.46 | 0.009 | | -0.61 | 0.001 |  |  | -0.33 | 0.07 | -0.63 | <0.001 | | -0.21 | 0.15 | -0.34 | 0.06 | -0.54 | 0.004 | -0.42 | 0.009 |  |  | -0.36 | 0.02 | -0.59 | | 0.002 |
| **Ophthalmological characteristics** | | | | | | | | | | | | | | | | | | | | | | | | | | | | | |
| **Binocular habitual visual acuity (LogMAR)** | -0.83 | 0.12 | -2.41 | <0.001 | | -0.58 | 0.29 | -0.60 | 0.06 | -1.51 | 0.008 | -0.99 | 0.05 | | -2.19 | <0.001 | -1.37 | 0.01 | -0.70 | 0.21 | -2.44 | <0.001 | -2.01 | 0.01 | -1.36 | 0.003 | -2.49 | | <0.001 |
| **Glasses wearing (yes/no)** |  |  |  |  | |  |  |  |  | 0.32 | 0.03 |  |  | |  |  |  |  |  |  |  |  |  |  |  |  |  | |  |
| **Lens status^*^** |  |  |  |  | |  |  |  |  |  |  |  |  | |  |  |  |  |  |  |  |  |  |  |  |  |  | |  |
| Cataract/clear lens | 0.10 | 0.59 | -0.44 | 0.01 | | 0.009 | 0.96 |  |  | -0.16 | 0.39 |  |  | |  |  |  |  |  |  | 0.09 | 0.56 | 0.01 | 0.95 |  |  |  | |  |
| Pseudophakia/clear lens | -0.05 | 0.81 | -0.39 | 0.10 | | 0.18 | 0.48 |  |  | -0.10 | 0.64 |  |  | |  |  |  |  |  |  | -0.03 | 0.90 | -0.26 | 0.35 |  |  |  | |  |
| **Eye operation** |  |  | 0.16 | 0.27 | | -0.02 | 0.91 |  |  |  |  |  |  | |  |  | -0.33 | 0.001 |  |  | -0.06 | 0.64 | -0.25 | 0.14 | -0.32 | <0.001 |  | |  |
| **Higher IOP** |  |  |  |  | |  |  |  |  | 0.05 | 0.01 |  |  | | 0.03 | 0.08 |  |  |  |  |  |  |  |  |  |  |  | |  |
| **Disease status of glaucoma** | | | | | | | | | | | | | | | | | | | | | | | | | | | | | |
| **Disease duration (months)** |  |  |  |  |  | |  |  |  |  |  | -0.001 | 0.04 | | <0.001 | 0.98 | <0.001 | 0.98 | <0.001 | 0.84 | <0.001 | 0.88 |  |  |  |  | <0.001 | | 0.29 |
| **Numbers of current glaucoma drug** |  |  |  |  |  | |  |  |  | -0.15 | 0.24 | -0.06 | 0.59 | |  |  |  |  | 0.03 | 0.78 |  |  |  |  |  |  | -0.06 | | 0.63 |
| **Dosing frequency of glaucoma medication (times/day)** |  |  |  |  | -0.12 | | 0.02 |  |  | 0.01 | 0.90 | -0.14 | 0.16 | | -0.07 | 0.09 | -0.07 | 0.15 | -0.17 | 0.11 | -0.10 | 0.03 |  |  |  |  | -0.10 | | 0.36 |
| **R-squared** | 0.24 | | 0.15 | | 0.13 | | | 0.07 | | 0.15 | | 0.18 | | | 0.13 | | 0.09 | | 0.11 | | 0.17 | | 0.20 | | 0.12 | | 0.18 | | |
| COPD, chronic obstructive pulmonary disease; IOP, intraocular pressure; LogMAR, logarithm of the minimum angle of resolution; NEI VFQ-25, National Eye Institute Visual Function Questionnaire  This model was adjusted for age, sex, binocular visual acuity, and other factors regarding socioeconomic data and ophthalmologic characteristics that attained statistical significance during univariable analysis. The factors that did not have statistical significance in the univariable analysis were not analyzed in the regression model, and the columns were empty.  ^*^Lens status: “Cataract” means cataract in at least one eye. “Pseudophakia” means pseudophakia in at least one eye. | | | | | | | | | | | | | | | | | | | | | | | | | | | | | |
